# Preclinical Diagnosis of Type 1 Diabetes: Reality or Utopia

**DOI:** 10.3390/biomedicines13102444

**Published:** 2025-10-07

**Authors:** Tatyana A. Marakhovskaya, Dmitry V. Tabakov, Olga V. Glushkova, Zoya G. Antysheva, Yaroslava S. Kiseleva, Ekaterina S. Petriaikina, Nickolay A. Bugaev-Makarovskiy, Anna S. Tashchilova, Vasiliy E. Akimov, Julia A. Krupinova, Viktor P. Bogdanov, Tatyana M. Frolova, Victoria S. Shchekina, Ekaterina S. Avsievich, Valerii V. Gorev, Irina G. Rybkina, Ismail M. Osmanov, Irina G. Kolomina, Igor E. Khatkov, Natalia A. Bodunova, Vladimir S. Yudin, Anton A. Keskinov, Sergey M. Yudin, Pavel Y. Volchkov, Dmitry V. Svetlichnyy, Mary Woroncow, Veronika I. Skvortsova

**Affiliations:** 1 Federal State Budgetary Institution «Centre for Strategic Planning and Management of Biomedical Health Risks» of the Federal Medical and Biological Agency (Centre for Strategic Planning of the Federal Medical and Biological Agency), 119121 Moscow, Russia; 2 Federal State Budgetary Scientific Institution “Federal Research Center for Innovator and Emerging Biomedical and Pharmaceutical Technologies”, 125315 Moscow, Russia; 3 Moscow Center for Advanced Studies, Kulakova Street 20, 123592 Moscow, Russia; 4 Institute of Cell Biophysics, Russian Academy of Sciences, PSCBR RAS, Institutskaya Street 3, 142290 Moscow, Russia; 5 Moscow Clinical Scientific Center N.A. A.S. Loginov, Novogireevskaya Street, 111123 Moscow, Russia; 6 Bashliaeva Children’s Municipal Clinical Hospital, Moscow City Health Department, Geroev Panfilovtsev Street 4, 125373 Moscow, Russia; 7 Morozovskaya Children’s City Clinical Hospital, 4 Dobryninskiy Pereulok, 1/9, 119049 Moscow, Russia; 8 Faculty of Fundamental Medicine, Lomonosov Moscow State University, Leninskie Gory Street 1, 119991 Moscow, Russia; 9 The Federal Medical Biological Agency (FMBA of Russia), 117513 Moscow, Russia

**Keywords:** type 1 diabetes mellitus, prediabetes, GWAS, scRNA-seq, cfDNA, microRNA, cytokines, FTIR spectroscopy, HLA-haplotype, non-HLA SNPs, islet antigens autoantibodies, C-peptide, T1D-specific immune cells, islet-TCR, T1D specific vibrational bands

## Abstract

Type 1 Diabetes Mellitus (T1D) is an autoimmune disease characterized by the destruction of pancreatic *β*-cells, predominantly manifesting in childhood or adolescence. The lack of clearly interpretable biological markers in the early stages, combined with the insidious onset of the disease, poses significant challenges to early diagnosis and the implementation of preventive strategies. The applicability of classic T1D biomarkers for understanding the mechanisms of the autoimmune process, preclinical diagnostics and treatment efficiency is limited. Despite advances in next-generation sequencing (NGS) technologies, which have enabled large-scale genome-wide association studies (GWASs) and the identification of polygenic risk scores (PRSs) associated with T1D predisposition, as well as progress in bioinformatics approaches for assessing dysregulated gene expression, no universally accepted risk assessment model or definitive predictive biomarker has been established. Until now, the use of new promising biomarkers for T1D diagnostics is limited by insufficient evidence base. However, they have great potential for the development of diagnostic methods on their basis, which has been shown in single or serial large-scale studies. This critical review covers both well-known biomarkers widely used in clinical practice, such as HLA-haplotype, non-HLA SNPs, islet antigen autoantibodies, C-peptide, and the promising ones, such as cytokines, cfDNA, microRNA, T1D-specific immune cells, islet-TCR, and T1D-specific vibrational bands. Additionally, we highlight new approaches that have been gaining popularity and have already demonstrated their potential: GWAS, single-cell transcriptomics, identification of antigen-specific T cells using scRNA-seq, and FTIR spectroscopy. Although some of the biomarkers, in our opinion, are still limited to a research context or are far from being implemented in clinical diagnostics of T1D, they have the greatest potential of being applied in clinical practice. When integrated with the monitoring of the classical autoimmune diabetes markers, they would increase the sensitivity and specificity during diagnostics of early and preclinical stages of the disease. This critical review aims to evaluate the current landscape of classical and emerging biomarkers in autoimmune diabetes, with a focus on those enabling early detection—prior to extensive destruction of pancreatic islets. Another goal of the review is to focus the attention of the scientific community on the gaps in early T1D diagnostics, and to help in the selection of markers, targets, and methods for scientific studies on creating novel diagnostic panels.

## 1. Introduction

Type 1 diabetes mellitus (T1D) is an autoimmune disease that targets pancreatic *β*-cells, resulting in impaired endogenous insulin secretion. Unlike type 2 diabetes, T1D is characterized by a significantly earlier onset: according to the literature, onset under 20 is observed in 18% of patients, between 20 and 59 years for 64%, and after 60 years in the remaining 18%. On average, the age of onset is 29 years. According to estimates by the International Diabetes Federation, by 2040 the number of T1D patients worldwide will reach 13.5–17.4 million [1].

It is currently recognized that three stages precede the clinical diagnosis of type 1 diabetes. Stage 1, when an autoimmune response to islet cells can be detected by the presence of a set or subset of specific autoantibodies in the blood. Stage 2, when measurable dysglycemia occurs. Stage 3, when glucose disturbances meet clinical diagnostic criteria for diabetes [2]. In addition to the established stages, Stage 0 is also identified for individuals at high genetic risk of developing T1D, and Stage 4 corresponds to the completion of the autoimmune process and critical reduction or even complete depletion of *β*-cell mass [3,4]. The clinical presentation of T1D is heterogeneous, including differences in age of onset and residual insulin secretion. Furthermore, the relationship between islet autoantibody appearance (seroconversion), islet immune infiltration (insulitis), *β*-cell destruction, and diabetes progression is more ambiguous than initially assumed [5].

Therefore, a task of modern diabetology is the search for non-invasive markers characterizing Stages 1 and 2 of T1D progression to reveal predisposition to the disease (Stage 0). *β*-cell dysfunction during T1D progression develops asymptomatically. Diagnosis may be delayed by several months to over five years. For patients diagnosed with Stage 1 T1D, the risk of clinical disease is 35–50% within 5–6 years, with Stage 2, the risk increases to 75% [6]. The authors of the Consensus Guidelines for Monitoring Individuals with Positive Islet Autoantibodies at Stage 3 diabetes note the challenge in monitoring disease development among patients who are positive for only a single diabetes-specific islet autoantibody (IAb+). Since such patients generally have a lower risk, there is a significant chance of missing the onset of disease. The guidelines recommend that adults with one detected IAb+ should be monitored for their diabetic status every three years, as also done during screening for type 2 diabetes risk. For patients with multiple detected IAb+, if the duration of normoglycemia at the early stage of T1D is 5 years, metabolic monitoring every 2 years is considered sufficient [7].

Prompt assignment to a certain risk cohort is important for further immunotherapy aiming to prevent the progression of autoimmune processes, which might occur in the near future. However, despite a large number of studies, a specific risk panel for T1D has not been suggested yet. For patients in the early stages of T1D, the currently available periodic medical monitoring methods of glucose levels and education for diabetes symptom awareness do not provide a sufficiently effective approach for accurately diagnosing the onset and progression of the autoimmune process [7]. Therefore, in order to ensure the correct application of precision pathogenetic therapy for type 1 diabetes, there is a need for standardized classical comprehensive methods for monitoring and predicting T1D development, such as C-peptide measurements, islet autoantibody determination, and the creation of new classification models combining both classical and novel biomarkers. The development of next-generation sequencing (NGS) techniques allows for conducting large-scale genome-wide association studies (GWASs) and developing polygenic risk scores (PRSs) associated with various diseases and quantitative traits [8], as well as to analyze gene expressions, ligand–receptor, and intercellular interactions using additional transcriptome sequencing. The development of more reliable polygenic risk scores and integrative diagnostic models based on NGS and other approaches possesses high potential not only for discovering new mechanisms of the autoimmune process in T1D, but also for achieving greater accuracy in risk group identification, age-of-onset prediction, and, consequently, for optimizing monitoring schemes for T1D development in high-risk groups based on the putative disease phenotype.

One of the reasons for the possible lack of efficacy of the proposed diagnostic protocols is the absence of consideration of genetic heterogeneity of T1D—information on this phenomenon is currently being accumulated and analyzed. The heterogeneity of T1D clinical manifestations suggests the possibility of grouping patients into subtypes (endotypes). Current practice typically considers two endotypes of T1D. Initially, the concept of endotype was based on assessing immune cell infiltration of pancreatic islets. Researchers identified two endotypes: Type 1 with high CD20^+^ cell infiltration and more severe insulin metabolism disturbances manifesting before the age of 7, and Type 2 with low infiltration, manifesting after the age of 12 [9]. Subsequently, a partial association of these endotypes with autoantibody production profiles was demonstrated [5]. At present, some researchers are beginning to distinguish up to six T1D endotypes, each associated with a specific pattern of blood biochemistry, autoantibody profiles, genetic associations, and age of disease manifestation [10]. As evidence accumulates for the existence of multiple T1D endotypes, new disease mechanisms are increasingly considered, involving numerous diverse pathways of progression and pathogenesis that may underlie the development of different T1D phenotypes [11].

Due to the current high interest in the problem of T1D and the development and integration of sophisticated omics techniques, a large amount of data has been accumulated on potential biomarkers of this process. Up-to-date analysis of this data can help to estimate the prospects of the approaches to preclinical diagnostics of T1D. The purpose of this critical review is to analyze the current understanding of various diagnostic methods that can be used as biomarkers of autoimmune type 1 diabetes mellitus at the early stages, when destruction of the pancreatic islets has just begun or even before its initiation. The search for relevant publications was carried out using the PubMed database.

## 2. T1D Classic Biomarkers

This chapter reviews classical genetic, immune, and metabolic markers available for large-scale screening that are used in clinical practice for the diagnostics and risk prediction of T1D.

### 2.1. Assessment of Genetic Predisposition to T1D

Years of worldwide research into the role of innate factors allow for viewing type 1 diabetes mellitus as a polygenic disease. The lack of absolute concordance of T1D in monozygotic twins and the relatively low prevalence among close relatives underlines the complex interplay of specific genetic factors, as well as the contribution of environmental factors to the disease pathogenesis [5,12].

Human leukocyte antigen (HLA) genes account for approximately 40–50% of familial predisposition among diagnosed T1D cases [13]. The primary role of HLA molecules is the presentation of potentially foreign antigens to the immune system. It is assumed that one of the main triggers of an autoimmune response in T1D is a number of infectious agents whose protein determinants are similar to particular HLA molecules. In this case, the immune response to determinants of the infectious agent also triggers against complexes consisting of HLA molecules and autoantigens, which resemble the infectious determinants, thereby disrupting immune tolerance [14].

Classical T1D-associated variants are polymorphisms in the DR-DQ loci [15,16]. The DR3-DQ2 and DR4-DQ8 haplotypes are present in 90% of individuals with T1D [17]. High risks for being diagnosed with T1D by age 15 have also been found for the DR3/DR4DQB1*03:02 haplotype [18]. Additionally, it has been demonstrated that heterozygosity for DR3/DR4-DQ8 increases the risk of type 1 diabetes by more than 30-fold [13]. The DRB1*15:01–DQA1*01:02–DQB1*06:02 (DR15-DQ6.2), DRB1*15-DQB1*06:02 (DR15DQ6) haplotypes, on the other hand, are associated with reduced T1D risk [13,16]. According to Feil R. et al., most T1D patients are negative for the protective DR15-DQ6 haplotype [19]. The primary HLA haplotypes and their roles in T1D are presented in Table 1.

To improve the predictive capacity of genetic risk scores, genetic variants associated with T1D but not related to HLA are also being sought. Enormous progress in identifying these polymorphisms has been made due to GWAS, which analyzes patient genomes using case-control groups. In Table 2, several non-HLA SNPs associated with T1D development obtained from GWAS comparisons are presented.

Currently, it is assumed that the risk of developing T1D depends on a specific set of SNPs in the patient’s genotype. The estimated impact of genetic variants on human phenotype can be viewed using the Polygenic Score Catalog (PGS catalog), which is the largest open database of polygenic risk assessments developed by researchers from different countries [31] and is based on GWAS Catalog data [32]. For T1D, polygenic risk scores are available, with variant counts ranging from 37 (PGS000022) to 63,182 (PGS003993) (Table 3) [33]. These polygenic risk scores summarize the contribution of specific HLA haplotypes and non-HLA components. One of the most accurate ones is the GRS2 risk score by Sharp et al. demonstrating AUC = 0.93 [15]. The scale by Bonifacio et al. [34] also includes many non-HLA SNPs and represents a combined model of the risk scores by Winkler et al. and Oram et al. [35,36]. Interestingly, different risk scores may include different polymorphisms within the same gene (e.g., INS, IL2RA, and BACH2 contain distinct polymorphisms in the Sharp and Bonifacio scales), highlighting their overall significance in the development of autoimmune disorders. Currently, it can be argued that the developed risk scores allow stratification of donors by the type 1 diabetes development risk, but the actual value of F1-metrics in the task of identifying diabetics in many populations remains low, and the identification and inclusion of new genetic variants associated with type 1 diabetes in risk scores continues to improve their accuracy and remains an important area of research.

Another important direction is the search for associations between polymorphisms and specific T1D endotypes, and consequently, the prediction of T1D onset age. For example, the Finnish DIPP study showed that polymorphisms in the PTPN22 and STAT4 genes were characteristic of patients whose first antibody was specific to insulin (IAA-first), while ERBB3 polymorphism was specific for GADA-first [37]. The TEDDY study demonstrated that the combination of HLA-DR3 haplotype and a BACH2 variant leads to a GADA-first phenotype, while a BTNL2 variant leads to an IAA-first phenotype [38]. The TEDDY study also notes the need to track lifestyle-associated events influencing the manifestation of a particular phenotype.

About 80% of GWAS studies were conducted on individuals of European descent [32]; therefore, a significant part of existing PGS is based on SNPs characteristic of European populations that have ethnospecific frequencies different from other populations. Moreover, Europeans represent only 16% of the world population [39]. Nevertheless, it is possible to identify different ethnic groups with different susceptibilities to pathologies. Population differences among ethnicities may be a limitation in the clinical application of polygenic estimates, which reduces the generalizability of the PGS and its effectiveness in global use. Therefore, the accuracy of polygenic estimates of most of the currently developed PGSs for non-European individuals may be significantly lower compared to the accuracy of PGS studies conducted on European populations, i.e., the reliability of estimates depends on the training genomic data [39,40]. It is important to note the difficulty in assessing PGS transferability across ethnicities, which researchers will need to address in the development of genetic risk panels.

Thus, genetic factors contribute considerably to the development of T1D. The application of developed polygenic risk scores allows stratification of at-risk groups for T1D, helping to determine the need for further monitoring of these patients. However, not all PGSs have achieved high predictive power, and the search for specific markers remains relevant.

**Table 3:** PGS description for T1D according to the PGS Catalog.

PGS	Number of Variants	Source of Variant Associations (GWAS)	Development/ Training	PGS Evaluation	Original Genome	Reference Build
PGS000022	37	—	—	African: 33.3%; Hispanic or Latin American: 33.3%; European: 33.3%; 3 Sample Sets	NR	[41]
PGS000869	48	—	—	European, 29,652 individuals	hg19	[42]
PGS001817	825	—	European, 391,124 individuals	European: 37.5%; African: 25%; Eastern asian: 12.5%; Middle Eastern: 12.5%; South Asian: 12.5%; 8 Sample Sets	GRCh37	[33]
PGS002025	1068	—	European, 391,124 individuals	European: 37.5%; African: 25%; Eastern asian: 12.5%; Middle Eastern: 12.5%; South Asian: 12.5%; 8 Sample Sets	GRCh37	[33]
PGS003993	63,162	European: 89.3%; African: 7.1%; Additional Diverse Ancestries: 3.6%; 59,527 individuals (100%)	European: 100%; 804 individuals (100%)	European: 80%; South Asian: 20%; 5 Sample Sets	GRCh38	[43]
PGS004009	4031	European: 89.3%; African: 7.1%; Additional Diverse Ancestries: 3.6%; 59,527 individuals	European, 804 individuals	European: 80%; South Asian: 20%; 5 Sample Sets	GRCh38	[43]
PGS004020	6682	European: 89.3%; African: 7.1%; Additional Diverse Ancestries: 3.6%; 59,527 individuals	European, 804 individuals	European: 80%; South Asian: 20%; 5 Sample Sets	GRCh38	[43]
PGS004035	56,562	European: 89.3%; African: 7.1%; Additional Diverse Ancestries: 3.6%; 59,527 individuals	European, 804 individuals	European: 80%; South Asian: 20%; 5 Sample Sets	GRCh38	[43]
PGS004063	56,288	European: 89.3%; African: 7.1%; Additional Diverse Ancestries: 3.6%; 59,527 individuals	European, 804 individuals	European: 80%; South Asian: 20%; 5 Sample Sets	GRCh38	[43]
PGS004078	56,288	European: 89.3%; African: 7.1%; Additional Diverse Ancestries: 3.6%; 59,527 individuals	European, 804 individuals	European: 80%; South Asian: 20%; 5 Sample Sets	GRCh38	[43]
PGS004093	61,651	European: 89.3%; African: 7.1%; Additional Diverse Ancestries: 3.6%; 59,527 individuals	European, 804 individuals	European: 80%; South Asian: 20%; 5 Sample Sets	GRCh38	[43]
PGS004102	61,651	European: 89.3%; African: 7.1%; Additional Diverse Ancestries: 3.6%; 59,527 individuals	European, 804 individuals	European: 80%; South Asian: 20%; 5 Sample Sets	GRCh38	[43]
PGS004117	131	European: 89.3%; African: 7.1%; Additional Diverse Ancestries: 3.6%; 59,527 individuals	European, 804 individuals	European: 80%; South Asian: 20%; 5 Sample Sets	GRCh38	[43]
PGS004132	354	European: 89.3%; African: 7.1%; Additional Diverse Ancestries: 3.6%; 59,527 individuals	European, 804 individuals	European: 80%; South Asian: 20%; 5 Sample Sets	GRCh38	[43]
PGS004162	62,645	European: 89.3%; African: 7.1%; Additional Diverse Ancestries: 3.6%; 59,527 individuals	European, 804 individuals	European: 80%; South Asian: 20%; 5 Sample Sets	GRCh38	[43]
PGS004171	520	—	European, 200,000 individuals	European: 100%; 1 Sample Set	GRCh37	[44]
PGS004172	70	—	European, 200,000 individuals	European: 100%; 1 Sample Set	GRCh37	[44]
PGS004173	295	—	European, 200,000 individuals	European: 100%; 1 Sample Set	GRCh37	[44]
PGS004174	49	—	European, 200,000 individuals	European: 100%; 1 Sample Set	GRCh37	[44]
PGS004175	315	—	European, 200,000 individuals	European: 100%; 1 Sample Set	GRCh37	[44]
PGS004874	56,916	European: 89.3%; African: 7.1%; Additional Diverse Ancestries: 3.6%; 59,527 individuals	European, 404 individuals	European: 100%; 8 Sample Sets	GRCh37	[45]

### 2.2. Limitations and Prospects of T1D Genetic Screening

According to available data, genetic predisposition cannot be considered the sole determinant for the development of T1D. When identifying patients at high risk, it is important to remember that the assessment remains preliminary and not precise, as external factors that trigger or neutralize diabetes manifestation are not taken into account. It should also be considered that a high number of variants does not always guarantee greater accuracy, confirming the need for further refinement of polygenic risk scores and for identifying the optimal set of variants. Among first-degree relatives, the risk ranges from 3% to 5%, it is 8% among siblings, and may exceed 70% in monozygotic twins [46]. A family history of T1D significantly increases disease risk, but approximately 90% of newly diagnosed cases have no relatives with this diagnosis [47]. Differences in T1D onset are even observed among monozygotic twins, especially for those diagnosed after the age of 15. The risk of developing T1D if the father is affected is higher than if the mother is affected (6% versus 1%, respectively) [48]. These facts point to potential limitations of risk scores and suggest possible roles for epigenetic mechanisms and the need to integrate non-genetic factors into diagnostic models.

Nevertheless, the challenge of improving the sensitivity and specificity of polygenic scores by optimizing the utilized variant set may be addressed through progress in sequencing technologies and association studies. It is currently believed that the development of several autoimmune diseases is linked to specific genome loci. Distinctive patterns of genetic associations typical for certain autoimmune diseases are observed (Table 4). In particular, the major histocompatibility complex locus is associated with most autoimmune diseases [49,50]. For example, Alshiekh S. et al. found that patients with both T1D and celiac disease, T1D only, or celiac disease only, more frequently carried DRB4*01:03:01, DRB3*01:01:02, DRB3*02:02:01, or DRB4*01:03:01 compared to healthy controls [51].

Non-HLA component SNPs may also be linked to multiple autoimmune diseases. Gokuladhas S. et al. [49] conducted a GWAS of 18 autoimmune diseases, including T1D. Most SNPs (*n* = 1879) were associated with only one disease, and 186 SNPs were linked to two or more diseases, comprising 9% of all identified SNPs. For T1D, 18 shared loci were found with systemic lupus erythematosus [49]. In the work by Demela et al., GWAS results for nine autoimmune diseases were summarized, and three logical clusters were identified based on their associated polymorphisms. The first cluster included gastrointestinal diseases (Crohn’s disease, ulcerative colitis, primary sclerosing cholangitis), the second included systemic lupus erythematosus, T1D, and rheumatoid arthritis, and the third, allergic diseases. The study showed that in these clusters, changes occur at different nodes in the same immune pathways, and different immune cell patterns are involved in pathogenesis [50]. Together, these data indicate that autoimmune diseases can be grouped logically, and genetic factor patterns may direct the autoimmune process to specific organ systems.

It is important to note that most identified SNPs are located in non-coding regions of the genome and are likely involved in regulating the expression of immune-related genes [49]. One such example is the cytotoxic T-lymphocyte-associated protein 4 (CTLA-4) glycoprotein, present on the surface of T-helper cells, transmitting an inhibitory signal to T-lymphocytes [52], the increased expression of which is associated with protective effects against several autoimmune diseases, such as rheumatoid arthritis (RA), systemic lupus erythematosus (SLE), T1D, and juvenile idiopathic arthritis (JIA) [50]. Thus, delineating the specificity of SNPs significant for T1D and other autoimmune diseases, alongside searching for new polymorphisms characteristic of specific study populations, is a possible way to increase the predictive power of risk scores.

Table 4 presents SNPs, which are associated with several autoimmune diseases, including T1D, based on results from two large-scale genetic studies. Li Y.R. et al. conducted a meta-analysis of data from over 6035 children with ten distinct autoimmune diseases. The control group included 10,718 individuals without a history of autoimmune or immunemediated diseases [28]. In the study by Robertson C.C. et al., with 61,427 individuals participating, 143 genomic loci were identified, including nearly 60 independent non-HLA candidate genes associated with T1D and other autoimmune diseases [30].

**Table 4:** SNPs with confirmed simultaneous association with T1D and other autoimmune diseases.

SNP	Region	Gene	Autoimmune Disease	Source
rs6679677	1p13.2	PTPN22	THY, PSOR, T1D, JIA	[28]
rs62324212	4q27	IL21	THY, AS, CEL, CVID, UC, T1D, JIA, CD	[28]
rs706778	10p15.1	IL2RA	THY, AS, PSOR, CEL, T1D, JIA	[28]
rs1689510	12q13.2	SUOX	PSOR, T1D	[28]
rs2641348	1p12	NOTCH2	CD, T1D	[30]
rs78037977	1q24.3	FASLG	Asthma, vitiligo, allergic sensitization, T1D	[30]
rs7582694	2q32.2-q32.3	STAT4	SLE, hypothyroidism, CEL, RA, T1D	[30]
rs10213692	5q11.2	ANKRD55/IL6ST	Body fat percentage, T1D	[30]
rs212408	6q25.3	TAGAP	RA, CD, MS, T1D	[30]
rs10245867	7p15.2-p15.1	JAZF1	MS, CD, eczema, T1D	[30]
rs11033048	11p13	SLC1A2	Eczema, hay fever, MS, SLE, T1D	[30]
rs968567	11q12.2	FADS2	Vitiligo, T1D	[30]
rs911263	14q24.1	RAD51B	RA, T1D	[30]
rs1052553	17q21.31	MAPT	PBC, SLE, RA, T1D	[30]
rs10795791	10p15.1	IL2RA	RA, T1D	[49]
rs11203203	21q22.3	UBASH3A	RA, T1D, Vitiligo	[49]
rs12720356	19p13.2	TYK2	CRD, PSOR, T1D, UC	[49]
rs12927355	16p13.13	CLEC16A	MS, T1D	[49]
rs2076530	6p21.32	BTNL2	RA, AS, T1D	[53]
rs3129953	6p21.32	BTNL2	T1D, THY	[53]
rs887464	6p21.33	PSORS1C3	RA, AS, T1D, THY	[53]
rs12708716	16p13.13	CLEC16A	MS, Primary Billiary Cirrosis, T1D	[54]
rs2292239	12q13.2	ERBB3	T1D, allergic sensitization	[54]

Abbreviations: AS—ankylosing spondylitis, CEL—celiac disease, CD—Crohn’s disease, CRD—chronic respiratory disease, JIA—juvenile idiopathic arthritis, MS—multiple sclerosis, PBC—primary biliary cholangitis, PSOR—psoriasis, RA—rheumatoid arthritis, SLE—systemic lupus erythematosus, THY—autoimmune thyroiditis, T1D—type 1 diabetes mellitus, UC—ulcerative colitis.

To develop genetic panels for assessing T1D predisposition, both conserved and population-specific polymorphisms should be taken into account. Analysis of the associations between HLA-gene variants of different ethnic groups with T1D risk and age of onset revealed the presence of ethnicity-specific genetic markers (Table 5).

The search for ethnically specific polymorphisms in non-HLA genes remains relevant. For example, a meta-analysis showed that the -607C/A variant in the IL-18 gene is associated with T1D susceptibility in Asians, but not in Europeans [63], and FokI (rs2228570) and BsmI (rs1544410) polymorphisms of the vitamin D receptor (VDR) gene are associated with increased T1D risk in African and American populations, whereas in a mixed cohort (Asian, American, European, Australian, and African), the values were statistically insignificant [64]. In African Americans, on loci 1p22.1, variant rs190514104 (NC_000001.11.93145882G>A) was associated with an increased risk of T1D (OR = 2.9); however, the authors note the need for validation in an independent study due to the cohort’s heterogeneity [30]. Thus, populations have their own characteristic genetic profiles marked by specific susceptibility markers, which should be taken into account in T1D diagnostics.

**Figure 1:**
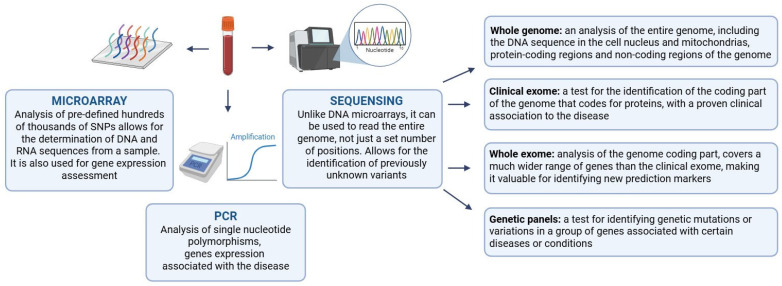
Methods for T1D diagnosing genetic predisposition.

Therefore, refining genetic risk scores for T1D development remains a pressing issue. Accounting for ethnic differences is among the most apparent routes for progress in this field. However, it should be remembered that methods of varying information volume are used to assess the contribution of innate factors: from examining targeted gene activity in high-risk patients to whole genome sequencing (Figure 1). The exome and genome analysis is preferable for research purposes to identify new significant polymorphisms. Due to high costs, these methods are difficult to apply routinely in clinical practice. Nevertheless, relatively inexpensive tests (DNA microarrays, genetic panels, created for T1D predisposition screening) can be applied in clinical diagnostic practice, based on the identified pathogenetically significant SNPs. In addition to direct risk assessment, genetic testing may predict the age of T1D onset. Accordingly, after risk estimation, an individual monitoring strategy can be offered and a therapeutic window for pathogenetic therapy identified. Integrating and identifying polymorphisms associated with specific age of onset will greatly improve the utility of risk scores and increase the effectiveness of early detection and treatment of T1D.

### 2.3. Markers of the T1D Autoimmune Process

#### 2.3.1. Autoantibodies to Islet Antigens

Specific autoantibodies to *β*-cell islet antigens accompany the autoimmune process in T1D; their presence in patients with hyperglycemia confirms the autoimmune etiology of diabetes. Currently, routine clinical practice includes the identification of nonspecific antibodies to a mixture of islet antigens (ICA, islet cell antibodies), as well as four main antigen-specific autoantibodies: to insulin (IAA), to tyrosine phosphatase (IA-2A, insulinoma-associated antigen-2), to glutamic acid decarboxylase (GADA), and to zinc transporter 8 (ZnT8A). Some combination of these autoantibodies is present at the onset of hyperglycemia in more than 90% of individuals with various T1D phenotypes. These autoantibodies are considered markers of the autoimmune process. Notably, the presence of multiple autoantibodies at Stage 2 in young individuals (<18 years) predicts progression to Stage 3 within 5–10 years. For example, antibodies against deamidated extracellular epitopes and unmodified tyrosine phosphatase IA-2ecA may serve as markers of the later, clinical stages of T1D (Stages 3 and 4), and their absence in serum indicates the lack of diabetogenic immune process in at-risk individuals [65]. Current guidelines state that the identification of any two types of autoantibodies is grounds for dynamic monitoring by an endocrinologist with periodic assessment of glycemic status [7]. According to the 2024 ISPAD guidelines, detection of a single autoantibody type warrants a monitoring protocol determined by age, while detection of two or more autoantibodies means more frequent monitoring points [66]. The identification of islet autoantibodies at Stages 1–3 of T1D may be an indication for antidiabetic therapy in some countries [7,67].

Certain associations have been established between specific autoantibody profiles, HLA haplotypes, and T1D phenotypes [68]. For example, it has been shown that in individuals with HLA-DR3, GAD-binding autoantibodies appear first, while in HLA-DR4 patients, insulin-binding autoantibodies predominate. It has also been shown that in those genetically predisposed to early T1D onset, the first autoantibodies usually appear at 1–2 years of age and are insulin-specific, whereas in individuals with later disease onset, the first autoantibodies are more often GAD or IA-2, detectable at the age of 4–5 years [69]. These observations make it possible to distinguish specific T1D endotypes with corresponding phenotypic, genetic, and immunological features [10].

With ongoing scientific research, new islet-specific autoantibodies are being gradually identified, which may offer prospects for preclinical diagnosis of T1D. For example, autoantibodies to the leucine variant of neuropeptide Y (NPY-LA) have been detected in some T1D patients. It is hypothesized that NPY-LA, via the MAPK pathway, may participate in the regulation of glucose homeostasis, inflammation, cell survival, ABC tranporter activity, and Ca^2+^ levels in β-cells [70].

It should be kept in mind that several years may pass between the detection of islet autoantibodies and the manifestation of the disease. Due to the association of autoantibody profile and HLA-haplotype, the prognostically significant parameter is the number of revealed antibodies rather than the first certain type of the revealed autoantibodies. Detection of 2 or more antibodies in the preclinical stage of T1D gives evidence on the initiation of the autoimmune process and the actual presence of Stages 1 or 2 of the disease in the subject. Thus, determining the spectrum of islet cell autoantibodies not only confirms the autoimmune nature of pancreatic involvement, but also allows identification of individuals at the earliest stages of autoimmune destruction, as well as optimization of current schemes of surveillance and early diagnosis of T1D.

#### 2.3.2. C-Peptide

C-peptide, a fragment of the proinsulin molecule cleaved during the early stages of insulin synthesis, is a marker of insulin production and is also widely used for diabetes diagnosis [71]. In T1D, a reduction in insulin synthesis occurs due to decreased *β*-cell mass, making C-peptide a marker correlated with the remaining *β*-cell mass. Nevertheless, due to its wide reference range and the high compensatory capacity of the pancreas, the use of C-peptide as an early marker of *β*-cell mass reduction is limited. For example, the study by Martinez M.M. et al. demonstrates the preservation of normal fasting C-peptide secretion in individuals with only one type of islet autoantibody (Single AAB group), and also shows the differences in fasting C-peptide levels between groups Single AAB and Multiple AAB for Finnish participants, while in the analogous groups of Swedish participants, the differences in this parameter were absent [72]. Thus, we can suppose that the decreased C-peptide level will occur only after the loss of a significant mass of *β*-cells, which renders the use of this marker irrelevant to preclinical T1D diagnostics.

Nonetheless, C-peptide can be used to assess residual insulin secretion in T1D patients. Januszewski A.S. et al. showed that adults with T1D onset more often had detectable C-peptide compared to those with childhood onset. The study also found that individuals with long-term T1D had higher C-peptide levels than those with a shorter disease duration. These findings confirm a faster rate of islet destruction in early-onset disease and suggest the possibility of some recovery of insulin secretion in T1D patients [73]. Subsequent studies have shown that this secretion may be provided by *α*-cells of pancreatic islets [74].

Several studies have highlighted the critical role of measuring C-peptide levels for determining diabetes type (autoimmune vs. T2D). Lower C-peptide concentrations are markers for T1D rather than T2D [75,76]. Another important application is the differential diagnosis of T1D and MODY diabetes resulting from mutations in the HNF1A/4A genes [77,78].

## 3. T1D Novel Biomarkers

The classical biomarkers described above are of limited value for preclinical T1D biomarkers, monitoring of disease progression, or treatment efficacy. This chapter is dedicated to the authors’ opinion on the most promising biomarkers, which could help to enhance the sensitivity and specificity at the early and preclinical stages of the disease if integrated with monitoring classical biomarkers. Currently, the majority are not used in clinical diagnostics of T1D, but their high potential for applicability has been demonstrated in individual or large-scale screening studies.

### 3.1. Cytokines in T1D Pathogenesis

Cytokines play an important role in organizing complex intercellular interactions between pancreatic *β*-cells and immune cells in diabetes involved in stimulation, regulation, and orchestration of the immune response, mediating insulitis and *β*-cell destruction.

Cytokines produced by immune and pancreatic cells affect the development and progression of T1D differently. IFN-*α*, IFN-*γ*, and TNF-*α* exert direct cytotoxic effects on *β*-cells via apoptosis induction, and they are among the main triggers for early T1D development [79,80,81]. IL-6, TNF-*α*, IFN-*α*, IL-17, and IL-21, which promote differentiation and function of diabetogenic immune cells, including Th1, Th17, CD8^+^ T cells, and NK cells, lead to T1D onset and progression (Table 6). In contrast, there is evidence that cytokines like IL-10, TGF-*β*, IL-5, IL-4, IL-2, IL-15, IL-33, and IL-35 restore immune tolerance and prevent *β*-cell damage. Due to pleiotropy, cytokines like IL-2 and IL-15 can activate both diabetogenic and regulatory immune cells. In addition, *β*-cells express high levels of cytokine receptors (e.g., IL-1R, IL-4R, and IL-22R) and demonstrate increased sensitivity to cytokine-induced apoptosis or regeneration [82]. Effective chemokines and their receptors can induce infiltration of various immune cells into pancreatic islets, where they attack *β*-cells. A systematic review of chemokine systems in the pathophysiology of T1D identified CCL5, CCL7, CXCL1, and CXCL9 as key players in pathogenesis [83].

During T1D manifestation, the overall immune response shifts toward pro-inflammatory processes, as demonstrated in various human and mouse tissues (Table 7). For example, a pro-inflammatory signature with increased frequencies of IFN-*γ*+TNF-*α*+ CD27−CD8^+^ circulating memory T-cells in the blood was observed in children with a recent T1D diagnosis (Stage 3) [98]. NOD mice with manifested diabetes showed elevated IL-12 and IL-6 and reduced IL-10 in gut cells (Table 7) [99]. Thus, the expression level of pro-inflammatory cytokines may serve as an important diagnostic marker for autoimmune process onset. For example, the IFN-1 signature is well described in multiple autoimmune diseases, including T1D [100]. Notably, the expression of type I IFN-induced genes is elevated in the blood of genetically at-risk children prior to the appearance of islet autoantibodies compared to controls but not in patients with established disease, indicating that innate type I IFN pathways are activated very early, at the pre-symptomatic Stage 0. The mechanistic role of interferon signaling in the induction of organ-specific autoimmunity like T1D remains unclear, although evidence suggests these cytokines may induce increased autoantigen presentation by islet cells, enhancing effector T-cell activation [81].

Currently, there are approaches to pathogenetic therapy based on inhibition of TNF*α* activity. The use of golimumab, an antibody to TNF-*α*, resulted in higher C-peptide levels compared to the control group 12 months after therapy administration [101]. The level of pro-inflammatory cytokines is typically high at the initial and destructive stages of T1D, when *β*-cell death occurs, and it is registered in patients with disease duration above 3 years. Particularly, elevated amounts of IFN-*γ*, TNF-*α*, IL-1*β*, and IL-6 were observed in children with T1D with duration in the range of 2.5 to 6.5 years [102].

**Table 7:** Cytokine levels in different T1D stages.

Cytokine	Level Compared to Control Group *	T1D Duration	Biomaterial	Comparison Group, N, Age	Reference
IL-10	↓	Preclinical stages	Small intestine cells	NOD mice, 4–6 weeks	[99]
IL-12	↑	Preclinical stages	Cells of the small and large intestines	NOD mice, 4–6 weeks	[99]
IL-6	↑	Preclinical stages	Colon cells	NOD mice, 4–6 weeks	[99]
IL-17	↑	Preclinical stages	Pancreatic cells	NOD mice, 4–6 weeks	[99]
IL1-RA	↓	2 weeks, Stage 3	Blood serum	100 patients up to 18 years old with newly diagnosed T1D	[103]
CXCL10	↑	4–9 weeks, Stage 3	Pancreatic islet cells	6 patients, 24–35 years old	[104]
CXCL10	↑	4–9 weeks, Stage 3	Pancreatic islet cells	NOD mice, 8 weeks	[104]
EGF	↑	4–16 years, Stages 3–4	Blood serum	52 patients with T1D, 8–18 years old	[105]
eotaxin/CCL11	↑	4–16 years, Stages 3–4	Blood serum	52 patients with T1D, 8–18 years old	[105]
MDC/CCL22	↑	4–16 years, Stages 3–4	Blood serum	52 patients with T1D, 8–18 years old	[105]
sCD40L	↑	4–16 years, Stages 3–4	Blood serum	52 patients with T1D, 8–18 years old	[105]
TGF-*α*	↑	4–16 years, Stages 3–4	Blood serum	52 patients with T1D, 8–18 years old	[105]
TNF-*α*	↑	4–16 years, Stages 3–4	Blood serum	52 patients with T1D, 8–18 years old	[105]
M-CSF	↓	1–23 years, Stages 3–4	Blood serum	25 patients (13 female and 12 male), 11–25 years old	[106]
IL-6	↓	1–23 years, Stages 3–4	Blood serum	25 patients (13 female and 12 male), 11–25 years old	[106]
CXCL1	↓	1–23 years, Stages 3–4	Blood serum	25 patients (13 female and 12 male), 11–25 years old	[106]
TGF-*α*	↓	1–23 years, Stages 3–4	Blood serum	Of these, 13 are female	[106]
IL-1*α*	↓	1–23 years, Stages 3–4	Blood serum	Of these, 13 are female	[106]
IL-4	↓	1–23 years, Stages 3–4	Blood serum	Of these, 13 are female	[106]
IL-13	↓	1–23 years, Stages 3–4	Blood serum	Of these, 13 are female	[106]
IL-22	↓	1–23 years, Stages 3–4	Blood serum	Of these, 13 are female	[106]
MIP-1*α*	↓	1–23 years, Stages 3–4	Blood serum	Of these, 13 are female	[106]
CCL5 (RANTES)	↓	1–23 years, Stages 3–4	Blood serum	Of these, 13 are female	[106]
MIP-3	↓	1–23 years, Stages 3–4	Blood serum	Of these, 13 are female	[106]
IL-22	↑	1–23 years, Stages 3–4	Blood serum	Of these, 12 are male	[106]
EGF	↑	1–23 years, Stages 3–4	Blood serum	Of these, 12 are male	[106]
PDGF-AB/BB	↑	1–23 years, Stages 3–4	Blood serum	Of these, 12 are male	[106]
IL-12	↑	8.0 ± 6.0 years, Stage 4	Blood serum	29 patients with T1D without microvascular complications, 21.5 ± 11.0 years old, receiving insulin and antihypertensive drugs	[107]
IL-33	↑	8.0 ± 6.0 years, Stage 4	Blood serum	29 patients with T1D without microvascular complications, 21.5 ± 11.0 years old, receiving insulin and antihypertensive drugs	[107]
IL-4	↑	8.0 ± 6.0 years, Stage 4	Blood serum	29 patients with T1D without microvascular complications, 21.5 ± 11.0 years old, receiving insulin and antihypertensive drugs	[107]
IL-10	↑	8.0 ± 6.0 years, Stage 4	Blood serum	29 patients with T1D without microvascular complications, 21.5 ± 11.0 years old, receiving insulin and antihypertensive drugs	[107]
IL-17	↑	8.0 ± 6.0 years, Stage 4	Blood serum	29 patients with T1D without microvascular complications, 21.5 ± 11.0 years old, receiving insulin and antihypertensive drugs	[107]
IL-9	↑	8.0 ± 6.0 years, Stage 4	Blood serum	29 patients with T1D without microvascular complications, 21.5 ± 11.0 years old, receiving insulin and antihypertensive drugs	[107]
IL-12	↑	8.0 ± 6.0 years, Stage 4	Blood serum	96 patients with T1D complicated by retinopathy and nephropathy, 29.0 ± 15.2 years old, receiving insulin and antihypertensive drugs	[107]
IL-33	↑	8.0 ± 6.0 years, Stage 4	Blood serum	96 patients with T1D complicated by retinopathy and nephropathy, 29.0 ± 15.2 years old, receiving insulin and antihypertensive drugs	[107]
IL-4	↑	8.0 ± 6.0 years, Stage 4	Blood serum	96 patients with T1D complicated by retinopathy and nephropathy, 29.0 ± 15.2 years old, receiving insulin and antihypertensive drugs	[107]
IL-10	↑	8.0 ± 6.0 years, Stage 4	Blood serum	96 patients with T1D complicated by retinopathy and nephropathy, 29.0 ± 15.2 years old, receiving insulin and antihypertensive drugs	[107]
IL-17	↑	8.0 ± 6.0 years, Stage 4	Blood serum	96 patients with T1D complicated by retinopathy and nephropathy, 29.0 ± 15.2 years old, receiving insulin and antihypertensive drugs	[107]
IL-9	↑	8.0 ± 6.0 years, Stage 4	Blood serum	96 patients with T1D complicated by retinopathy and nephropathy, 29.0 ± 15.2 years old, receiving insulin and antihypertensive drugs	[107]

* ↑ is increase, ↓ is decrease.

Other candidates for the role of serum biomarkers predictive of impending clinical T1D include osteopontin (OPN), a cytokine-like phosphoprotein involved in many physiological and pathological processes, including autoimmunity and potentially even immune processes that occur early before disease onset. Elevated OPN levels are observed in pancreatic lymph nodes of NOD mice during prediabetic stages [108]. In Opn-null mice, the absence of OPN accelerated T1D development, suggesting a protective role of this protein in insulin-producing islet cells [109]. Using expression-based genome-wide association study (eGWAS) technology, Jia X. et al. found that children with newly-diagnosed T1D had increased OPN and low IL1-RA levels in their serum compared to healthy controls [103].

Thus, complex expression panels of cytokine networks correspond differentially to the phases of T1D initiation and progression. However, the clinical use of cytokines as serum biomarkers is limited because their synthesis reflects not only diabetogenic processes but also responses to tissue injury, inflammation, or immune-associated diseases. Nevertheless, in established T1D, blood cytokine profile changes, in addition to carbohydrate metabolism disturbances, may serve as important markers of disease progression rate. Analysis of cytokine networks involved in T1D pathogenesis could be a successful strategy for both diagnosis and estimation of immunotherapy efficiency of this disease. The search for specific cytokines associated with different stages of T1D, including the early ones, is still in progress. For example, prospective studies have shown that the level of cytokines (IFN-*γ*, TNF-*α*, IL-1*β*, IL-4, IL-6, IL-10, IL-13, IL-18, IL-21, IL-35, and IL-37) was significantly higher in T1D subjects [110], and it was maintained for 5 years, followed by a further decrease of these pro-inflammatory mediators [111]. Other prospective T1D studies showed that lower levels of IL-10, IFN-*γ*, and IL-1R1 at the moment of diagnosis establishment are related to remission [112], but their revelation is not sufficiently effective for the use as markers for diagnostics of full remission at the stage of postprandial hyperglycemia in T1D [113]. A hypothesis on the existence of specific T1D-associated cytokine profiles is confirmed by a high degree of correlation of cytokine and IAA content. For example, the association of IL-21 level with ICA and that of IL-10 with GADA was revealed in the blood of T1D patients from Saudi Arabia [102]. Currently, serum level of pro-inflammatory cytokines shows a general inflammation process in T1D patients, but it cannot be used for early diagnostics and monitoring disease progression [113]. However, a thorough search of T1D-specific cytokine panels in prospective studies might identify a crucial component of the signature of circulating biomarkers of autoimmune diabetes.

Besides assessing the pro-inflammatory status of the immune system, cytokine secretion monitoring may become an effective biomarker of response to pathogenetic therapy [114,115]. However, at present, there are no established recommendations or protocols for cytokine profile monitoring in high-risk T1D groups described in the literature, which could be addressed in the future as part of biomarker studies for anti-cytokine anti-inflammatory therapy or for elaboration of diagnostic scales.

### 3.2. Circulating Cell-Free DNA as a Biomarker for T1D Diagnosis

Circulating cell-free DNA (cfDNA) is one of the markers of cell destruction arising from processes such as necrosis, apoptosis, cell lysis, or NETosis. It has been shown that cfDNA is present in a wide range of extracellular fluids such as plasma, serum, urine, saliva, and cerebrospinal fluid, in the form of structures like microvesicles, microparticles, apoptotic bodies, exosomes, histone complexes, and virtosomes [116]. Discovered in 1948, cfDNA was long regarded as a marker associated with systemic lupus erythematosus [117]. Today, changes in blood serum cfDNA concentration have been demonstrated in pregnancy pathologies, oncological, neurological, and autoimmune diseases, as well as during inflammatory processes and physical activity [118,119,120,121].

The destruction of *β*-cells in T1D is also accompanied by the release of cfDNA fragments into the bloodstream, followed by their excretion from the body. Since the epigenome is unique for each cell type, detection of DNA with specific epigenetic marks in serum can identify DNA fragments from the target cells. CpG sites are often unmethylated in cells actively transcribing a gene; thus, hypomethylated sequence stretches within the insulin gene promoter can be used to detect DNA from destroyed islet cells [122]. The presence of unmethylated circulating cfDNA from the insulin promoter in the blood of newly diagnosed T1D patients has also been confirmed by other studies [123]. Experiments in NOD mice have verified that this method can detect *β*-cell death even before blood glucose levels rise [124,125,126].

The level of cfDNA in T1D patients varies depending on disease duration. For example, it is known that neutrophils infiltrate the pancreas long before T1D symptoms occur, driving the initiation and progression of the disease, including through release of neutrophil extracellular traps (NETs) composed of modified DNA fragments during NETosis. Patients with newly diagnosed T1D show a pronounced increase in circulating cfDNA markers of NETosis, while in those with a disease history of over 5 years (mean 7.6 years), marker levels in the blood are lower, and for disease durations over 16 years in men, cfDNA (NET) levels are similar to those of healthy controls. These data suggest that neutrophil involvement and activation, including cfDNA formation, are especially intense at disease onset but return to baseline over time [127,128]. This phenomenon requires further study, since the sample sizes in both cited studies (146 and 14 T1D patients, respectively) were small, and the data need clarification.

cfDNA can also be used to assess the survival of pancreatic islets. It has been shown that cell death during islet transplantation is accompanied by increased cfDNA one hour and 24 h after islet infusion. High levels of cfDNA 24 h post-transplant in patients correlate with increased insulin levels and lower stimulated C-peptide levels over one month, which can be used to assess graft viability and plan repeated islet infusions [129,130].

However, the application of cfDNA in T1D diagnostics faces some limitations. One of such limitations is a short half-elimination period of cfDNA, up to 90 min [16], which can lead to failure to detect important changes. A possible solution is auxiliary analysis measuring the levels of differentially methylated INS and CHTOP in longitudinal cohort studies [131].

Another important aspect is non-specificity of hypomethylation of insulin gene in *β*-cells. Death of skeletal muscle cells and adipose tissue also leads to hypomethylation of this gene [132]. In this regard, the cell type of the analyzed sample must be taken into consideration, but it brings additional complications into procedure and increases its invasiveness. Technical limitations are related to the features of the applied methods of cfDNA level measurements, such as digital PCR and targeted bisulfite sequencing. To overcome these barriers, innovative methods of bioinformatic processing should be applied, and minimal requirements for biological sample quality should be satisfied before the clinical implementation. Usmani-Brown S. et al. have proposed an improved method for detecting circulating cfDNA with *β*-cell-specific epigenetic modifications of the INS gene in blood serum [124]. Such technology is highly specific for identifying metabolic status changes in *β*-cells before their destruction and represents the most promising direction for preclinical T1D diagnosis in at-risk individuals, though further refinement is needed.

Key methods of cfDNA methylation analysis include analytical techniques based on its digestion by methyl-sensitive restriction enzymes followed by PCR (MSRE-PCR) or sequencing, methods based on bisulfite DNA conversion followed by PCR (MS-PCR) and sequencing, affinity capture of the methylated DNA with subsequent analysis, and liquid chromatography combined with tandem mass spectrometry (LC-MS/MS) [133,134]. The most promising methods combining advantageous costs, sensitivity, and accuracy, include targeted bisulfite capture of DNA (or enriched DNA loci carrying the gene of interest) followed by sequencing or digital PCR analysis. This could lead to the highest precision of the analysis due to exclusion of systematic errors accompanying classical methods having lower sensitivity and high variability of measurement ranges.

Low reproducibility is related to the differences in approaches to cohort assignment of the subjects, insufficient diagnostics and characterization of the groups, intragroup differences in immunophenotypes [135], ethnicity, lack of consideration of accompanying diseases, differences in nutrition type, and time of biological sample acquisition. There is a noticeable inconsistency in the findings regarding the detection of cfDNA in patients with T1D [130,136,137,138]. This can be explained by the fact that a significant portion of *β*-cells are destroyed during the preclinical stage, while patients are typically examined at later stages of the disease. A solution could be in standardizing the workflows and study trajectories confirming the reproducibility of results.

CfDNA analysis is a non-invasive and inexpensive method, so numerous studies are focused on its broad clinical application. However, despite its high sensitivity, this approach can yield false-negative results and thus requires improvement [137]. The lack of standardized methodologies further complicates the picture, especially since research on cfDNA as an early marker for T1D has only recently begun to develop actively (a PubMed search using the keyword “T1D cfDNA” yields 14 publications from 2016 to 2025).

### 3.3. T1D MicroRNA Expression Profile

At present, the role of short RNA transcripts, such as small noncoding RNA (microRNA, miRNA), serving as global regulators of gene expression via post-transcriptional silencing, is being actively explored in T1D pathogenesis [139]. A PubMed search for “T1D microRNA” revealed 148 publications spanning 2008–2025, with a peak in 2024.

MicroRNA regulates *β*-cell gene expression in the pancreas, suppression of insulin gene expression, and stimulation of the autoimmune response against *β*-cells. The literature mentions miR-21, miR-132-3p, miR-101-3p, miR-148b-3p, and miR-1275 as associated with *β*-cell dysfunction [140,141]. For instance, increased miR-21 expression inhibits transcription of the apoptosis regulator Bcl-2, promoting *β*-cell apoptosis during T1D development. In animal models of T1D and cytokine-treated cells and islets, miR-21 expression greatly increases, resulting in reduced cell number and viability and increased caspase 3 levels [142]. Remarkably, some microRNAs can directly influence the degradation of the pancreatic islet region during T1D development. miR-200 and miR-204 are involved in the TXNIP/miR-200/ZEB1/E-cadherin pathway, which activates the expression of TXNIP (thioredoxin-interacting protein), also known as TBP2, a well-known diabetes biomarker. Increased TXNIP expression leads to increased miR-204 expression, which subsequently initiates the ZEB1 (zinc finger E-box-binding homeobox 1)–E-cadherin signaling cascade, causing *β*-cell apoptotic death. Thus, miR-200 and miR-204 are the initial triggers of apoptotic pathways in *β*-cells and can be used as biomarkers of progression from prediabetes to type 1 diabetes in the early stages, when islet destruction has just begun [143,144]. In addition, aberrant microRNA expression is observed in T1D, possibly due to the sensitivity of the miRNA profile to hyperglycemia [145,146], but as predictors of T1D—e.g., miR-200a-3p and miR-16-5p—microRNAs are more effective than glucose measurement [147]. Both upregulated and downregulated microRNAs have been identified in the biological fluids and tissues of patients with T1D [148]. Increased plasma expression of miR-23a-3p, miR-23b-3p, miR-24-3p, miR-27a-3p, miR-27b-3p, miR-21-3p, miR-29a-3p, and miR-424-5p is a wellestablished expression biomarker of T1D progression versus healthy controls. Heightened expression of this regulatory microRNA signature positively correlates with osteoprotegerin and negatively with soluble CD40 ligand, resistin, myeloperoxidase, and soluble TNF receptor in children with confirmed diagnosis. Two independent pediatric cohorts have shown the possibility of diagnosis and prediction of disease progression within 12 months before clinical manifestation, making regulatory RNAs currently among the most accurate predictive biomarkers [149]. To date, it has been shown that peripheral microRNA expression levels (such as miRNA-125b, miRNA-144, let-7, miRNA-155, miRNA-29, miRNA-133a, and miRNA-7) in blood and urine can distinguish T1D patients from healthy subjects with sufficient accuracy [148].

Interestingly, highly specific microRNA biomarkers of the autoimmune diabetogenic process have been found in pancreatic islets: for example, miRNA-204, released from dying *β*-cells at early disease stages. Unlike other microRNAs, miRNA-204 is specific for T1D as it is not detected in T2D or other autoimmune diseases. In lactating women with T1D, elevated plasma levels of hsa-miR-127-3p, hsa-miR-146a-5p, hsa-miR-26a-5p, hsamiR-24-3p, and hsa-miR-30d-5p have been found, which are connected with inflammatory responses mediated by chemokines and cytokines [150]. The same authors previously reported differences in miRNA levels in breast milk from mothers with type 1 diabetes and from healthy controls. In particular, women with T1D had increased breast milk levels of hsa-miR-4497, hsa-miR-1246, hsa-miR-133a-3p, hsa-miR-3178, hsa-miR-1290, and hsa-miR320d and lower levels of three miRNAs: hsa-miR-518e-3p, hsa-miR-629-3p, and hsa-miR200c-5p [151]. Moreover, technologies such as small RNA-Seq and real-time qPCR-rt have enabled the identification of a panel of 47 human islet microRNAs, which holds potential for early clinical detection of presymptomatic T1D [152].

Since microRNAs are found in a variety of biological fluids, including blood, urine, saliva, CSF, milk, and amniotic fluid, and are more stable than other RNA types, they are convenient objects for analysis as T1D biomarkers [151,153,154]. For example, Osipova J. et al. showed that miR-21 is significantly higher in urine and plasma from T1D children aged 6–18 years with disease duration of one year. They also identified increased miR-210 levels in plasma and urine, and low urinary miR-126, which negatively correlated with average glycated hemoglobin, while circulating plasma miR-126 levels matched those of the controls [155].

Circulating miRNA levels of miR-21 and miR-210 were significantly up-regulated in the plasma and urine of the type 1 diabetic patients. Urinary miR-126 levels in diabetic patients were significantly lower than in age- and gender-matched controls and negatively correlated between the patient’s glycated hemoglobin mean and miR-126 concentration value.

Thanks to the systemic stability of microRNAs compared to other types of RNA, they serve as convenient and reliable biomarkers in laboratory research [153]. For instance, the exRNAQC study represents the largest and most comprehensive assessment of pre-analytical factors affecting extracellular transcriptomes based on up-to-date sequencing [156]. In practice, the most commonly used methods are RT-PCR [147] or RTqPCR [157]—accessible and widely adopted techniques—or a more costly option of whole miRNome sequencing [158]. However, working with microRNAs at the pre-analytical stage presents certain challenges, which are nonetheless effectively addressed.

## 4. New Approaches and Methods in T1D Diagnosis

**Figure 2:**
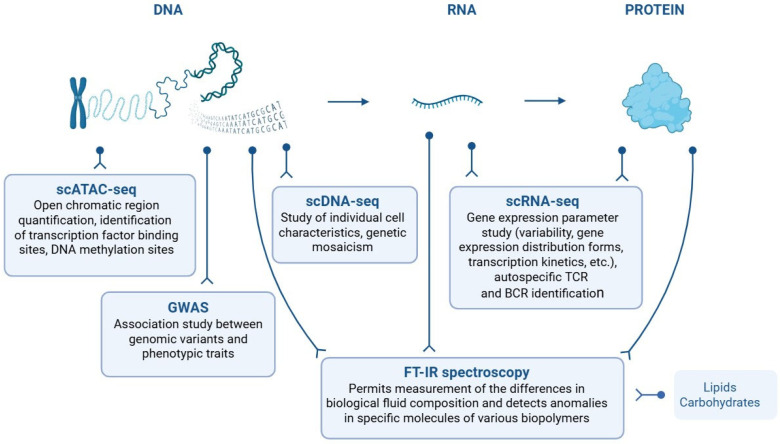
New approaches and methods in T1D detection and development of diagnostic markers.

T1D is a polygenic disease with high heterogeneity of genetic and environmental risk factors and variability in clinical manifestations. Importantly, genetic predisposition is only one part of a broader picture, behind which are many environmental and lifestyle factors. The current level of advancement in biomedicine—including the ability to assess the contributions of individual immune or islet cell populations to disease risk or T1D autoimmunity, and to understand the roles of complex gene interactions—has enabled the discovery of new biomarkers for disease risk or autoimmune diabetes progression. A key aspect of modern T1D diagnostics is the use of transcriptomics—gene expression analysis—to reveal which genes are activated in response to various signals. The discovery of new biological markers will require improvement, cost reduction, and simplification of experimental methods and adaptation of sample processing protocols for routine clinical use. Here, we have reviewed new approaches that are just gaining in popularity but have already demonstrated their promise for T1D diagnosis (Figure 2).

### 4.1. Cellular Markers of T1D

#### 4.1.1. Novel T1D-Specific Immune Cell Markers Accrued by Single-Cell Transcriptomics

In T1D diagnosis, sample composition assessment, regulatory network analysis, and intercellular communication assessment should take into account the gene expression profiles of individual immune and insulin-producing cells. In recent years, the study of individual cell transcriptomes via high-throughput RNA sequencing has become widespread. scRNAseq allows for the mass determination of gene expression at the single-cell level [159,160]. This approach is also essential to assess the response of specific cell populations to stimuli (treatment, environment, etc.) and enables the identification of process key genes and cell trajectories during these changes [161].

ScRNA-seq studies in T1D diagnostics provide detailed, dynamic expression profiles of peripheral blood immune cells or pancreatic cells at the single-cell level and can identify T1D gene biomarkers. The scRNA-seq approach has also greatly improved our understanding of intercellular interactions between resident and infiltrating immune cells, which are crucial in T1D development [162].

In children with newly diagnosed T1D, reduced expression of the genes PTPN6, TGFB, and TYROBP has been found in blood-derived dendritic cells, whose products participate in negative regulation of the immune response [163]. Dendritic cells express MHC class I and II molecules, presenting antigen fragments to T-cells; without antigen-presenting cells, T-cells cannot recognize an antigen. Therefore, reduced activity of the genes PTPN6, TGFB, and TYROBP contributes to enhanced autoimmune response in T1D. Analysis of differentially expressed genes in pancreatic immune cells identified REG1B, REG1A, INS, REG3A, and IL-32 as highly expressed in T1D [164].

In an experiment in rats, C1QB and NKG7 were shown to be associated with increased macrophage and CD8^+^ T-cell populations in the pancreas, respectively, resulting in *β*-cell injury [165].

ScRNA-seq is closely related to scATAC-seq, which analyzes DNA from individual cell nuclei to identify accessible chromatin regions and thus predict which genes are being expressed in each cell. Combining scATAC-seq with scRNA-seq has helped identify numerous cis-regulatory elements (CREs) used in T-cells and *β*-cells, which have genetic variants linked to T1D susceptibility [29]. For example, CREs that regulate CTLA4 and CCR7 expression in T-cells were found to have T1D-associated variants [30].

Analysis of the transcriptome signature and chromatin state of antigen-specific T-cells in blood could serve as a promising biomarker for T1D diagnosis. This is reflected in increased research interest: a PubMed search for “scRNA-seq T1D” yields 39 publications from 2017–2025, with a publication peak in 2024 (19 articles). Nevertheless, further research is necessary before these biomarkers can be introduced into clinical practice.

#### 4.1.2. Antigen-Specific T-Cells as T1D Biomarkers

The application of antigen-specific T-cells as T1D biomarkers has some limitations. First, there is a limited number of available antigen-specific TCR datasets from people with and without T1D that can be used for accurate matching of T1D-specific TCR clonotypes [166]. Second, it is known that with age the diversity of variable TCR-*β* repertoires decreases, while clonal expansion of T-cells with *β*-cell autoantigen-specific receptors increases [167]. This limits the value of antigen-specific cells for preclinical T1D diagnostics using scRNA-seq given the low frequency of T1D-specific TCR clonotypes in blood. This may be overcome by sample enrichment prior to sequencing, targeted sequencing of TCRs with preferred V gene segments, 5′RACE TCR-seq, or deep paired TCR repertoire sequencing (TIRTL-seq) [168]. Combining scRNA-seq with targeted techniques, developing open-access T1D-specific TCR databases, and creating new bioinformatics approaches for high throughput sequencing data will help standardize the use of antigen-specific T-cells as T1D biomarkers [169].

In addition to microRNA sequencing in blood, testing for islet-specific autoreactive T-cells is currently difficult to implement in routine clinical practice, primarily because of their high expense, the complexity involved in interpreting results, and the absence of standardized protocols.

### 4.2. Molecular Markers of T1D

#### T1D-Specific Key Molecular Markers, Accrued by FTIR Spectroscopy of Biological Fluids Combined with Deep Learning

Serological, cytological, genetic, and transcriptomic biomarkers are the mainstays of early T1D diagnosis, but their complexity, invasiveness, and interpretative ambiguity due to disease heterogeneity require new approaches. The simplest and least invasive method for early T1D diagnosis may be IR/FTIR spectroscopy screening of tear fluid, blood serum, saliva, or blood immune cells [170,171,172,173]. FTIR spectroscopy detects compositional differences in biological fluids and can identify anomalies in specific protein, lipid, nucleic acid, and other key molecular markers of T1D pathogenesis [174]. Recent studies have used deep machine learning (CART and XGBoost) in combination with FTIR spectroscopy, allowing this approach to compete with classic early diagnostic methods for various diseases, including T1D [175,176]. For example, for the XGBoost T1D diagnostic model, the specificity was 100.00% (25/25), the sensitivity was 85.00% (17/20), and the accuracy was equal to 93.33% (42/45) [175,176].

Using this approach, not only glucose but also other biomarkers—including T1Dand prediabetes-specific FTIR spectra—may be monitored. In saliva, FTIR spectra specific to T1D patients have been identified, with peaks in the 600–1300 cm^−1^ region (including a peak at 1076 cm^−1^ corresponding to a vibrational mode of DNA in the skeletal cisconformation), at 1403 cm^−1^ (symmetrical bending of *CH*_3_ methyl groups of proteins and *δ*s*CH*_3_ of collagen), and at 1451 cm^−1^ (asymmetric bending mode of *CH*_3_ methyl groups in proteins) [170]. As T1D progresses, both metabolism and the molecular signatures of immune cells change; thus, metabolic fingerprints of peripheral blood mononuclear cells (PBMCs) specific for different autoimmune diabetes stages are investigated. PBMC FTIR spectra in T1D patients versus controls also show peak shifts indicating extensive DNA rearrangement or fragmentation (1040–1280 cm^−1^), intensified glycolysis in certain PBMC subpopulations (1070 cm^−1^), and alterations in protein content and secondary structure (amide II band peak at 1545 cm^−1^ (dN–Hbond)) [173,177]. The absorbance band fluctuation with a maximum at 2956 cm^−1^ in PBMCs of T1D patients corresponds to –*CH*_3_ bonds in lipids and amino acid side chains associated with 2-deoxy-D-ribose-dependent oxidative phosphorylation of DNA and phospholipids in autoimmune diabetes [171,173]. This work showed an inverse AUC = 0.33 ± 0.096, N = 15 for a small number of participants in the study (up to 30 participants in a group), a stable trend in the prediction of T1D based on FTIR metabolic fingerprint data in the PBMC [171,173]. ATR-FTIR spectroscopy combined with principal component analysis/linear discriminant analysis (PCA-LDA) allowed for effective diagnosis of T1D and prediabetes in large patient cohorts (accuracy—97%; sensitivity—100%, 94%, and 91%; specificity—100%, 98%, and 98% in the control group, pre-diabetes group, and T1D group, respectively), demonstrating this approach’s promising clinical potential [172]. There is no doubt that the use of T1D-specific vibrational bands for clinical implementation is restricted by the absence of minimal technical requirements to the methodology of preanalytical and analytical stages, bioinformatic pipelines, and control checkpoints of external validations. Nevertheless, combining FTIR spectroscopy with deep learning is a new approach to early T1D diagnosis. Available screening assay by FTIR examination of immune cells from at-risk T1D patients may be promising for preclinical diagnosis. However, large-scale studies with more participants and robust stratification by clinical features, genetic risk, and T1D duration are required to obtain T1D-specific calibration spectra.

## 5. Discussion

**Figure 3:**
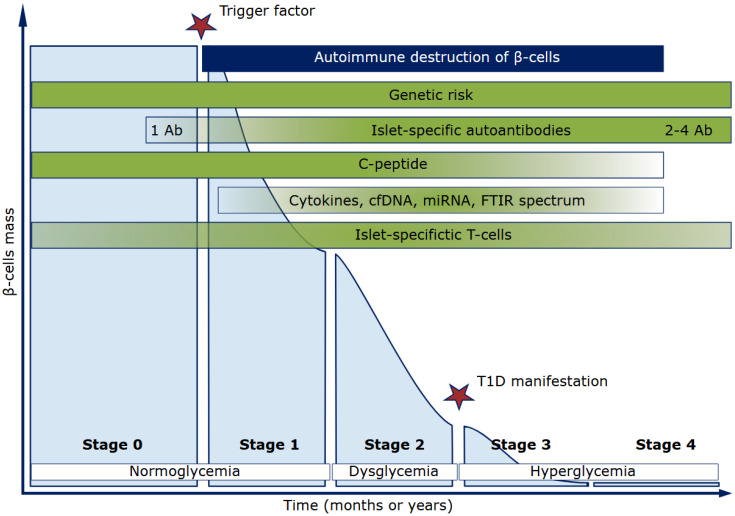
T1D marker representation at different stages of the disease.

The search for non-invasive markers to characterize T1D course or identify susceptibility to the disease is a vital challenge in modern diabetology. The main aims of preclinical T1D diagnosis are to determine a monitoring strategy for individuals at high T1D risk and define potential therapeutic windows for pathogenetic therapy. The biomarkers described here reflect current views on T1D pathogenesis and can be detected at different stages of disease progression (Figure 3). Developing universal methods for early T1D diagnostics remains a complex, multidisciplinary challenge.

Currently, three standard guidelines for T1D diagnosis and monitoring have been issued: the American Diabetes Association Consensus Guidance, the 2024 ISPAD Lisbon monitoring scheme, and the ADA2025 guidelines. According to the first guideline [7], the detection of a single autoantibody type serves as a viable reason for periodic screening, while the appearance of two or more autoantibodies is used to define stage 1 or 2 T1D based on glycemic status. The ISPAD2024 recommends tailor monitoring schemes to patient age and advises discontinuing follow-up in the absence of progression. ADA2025 recommendations call for monitoring individuals with a family history of T1D or other known genetic risks, and consider the presence of multiple autoantibodies as grounds for recommending pathogenetic therapy [178].

Some countries have screening programs for islet autoantibodies. The largest, TRIALNET TN01, enrolled 250,000 T1D relatives aged 3 to 45 years in the USA, Canada, Europe, and Australia, testing for ICA, anti-IA-2, GADA, and IAA [179]. The European INNODIA study included both relatives of T1D patients and the general population (IAA, GADA, anti-IA-2, ZnT8A) [180]. Both studies found comparable rates of healthy individuals with two or more detectable autoantibodies (2.5% and 2.6%, respectively), highlighting the importance of preclinical T1D diagnosis in high-risk groups. In addition, the ASK study in Colorado, testing 25,738 children aged 1–17, found that 0.52% were double-positive for IAA, GADA, anti-IA-2, ZnT8A, and tissue transglutaminase tTGA [179]. These studies show that in certain cohorts of healthy but at-risk individuals, active autoimmune processes are already present, creating appropriate grounds for pathogenetic therapy.

Thus, currently there is no method to reveal subjects with prospect of further T1D progression at Stage 0 (Table 8). Presently suggested genetic risk scales are the most promising approach to solve this problem, but they require enhancing their diagnostic specificity. Another option of “Stage 0”-diagnosis is monitoring the levels of diabetes-specific T-cell clones. Although technically challenging because these cells are scarce in peripheral blood, detection of clonal expansion may signal the need for anti-TCR or alefacept (LFA3-Ig) therapy [181,182]. We cannot exclude that further transcriptomic studies of immune cells will reveal cellular conditions precluding the onset of the autoimmune process. In this regard, the application of FTIR spectroscopy of biological fluids combined with deep learning is also very promising, not just allowing validation of subject assignment to T1D group, but also identifying subjects with prediabetic state. Nevertheless, only single cohort studies of T1D-specific vibrational bands are available, and additional standardization of the working processes, protocols, and research trajectories involving FTIR and considering the limitations of preanalytical, analytical, and postanalytical validation stages is required.

When discussing the antibody screening, we should note that the appearance of the first autoantibody does not always mean the onset of autoimmune degradation of the pancreas: many patients do not demonstrate further T1D progression at all. Based on the data presented in the previous chapters, we propose the following classification of biomarkers, which takes into account their application, prospects and level of readiness for implementation in clinical and preclinical practice for the diagnosis of type 1 diabetes mellitus, which is presented in Table 8. The table lists the most promising, in our opinion, biomarkers of the preclinical stages of T1D, limited only by the research context, biomarkers that are closer to practical application, but the use of which in preclinical diagnostics should be additionally confirmed and clearly standardized. Our review demonstrates that new markers of the autoimmune process(cfDNA, miRNA, cytokines, T1D-specific immune cells) (Table 8), which have yet to bridge the gap between research and practice, can potentially help in the determination of the triggering of *β*-cell destruction, which means that future studies in these directions are of great importance. Also, autoantibody detection, while revealing the existence and progression of the autoimmune process, does not clarify the mechanisms behind it, nor does it indicate which branch of the immune system should be targeted by inhibitory agents. Additional studies of new genetic, transcriptomic, and immune markers (Table 8) could determine T1D heterogeneity with improved accuracy in the future, which is important for target therapy selection. For implementation and monitoring of pathogenetic therapies—for example, anti-TNFa agents—may be useful to monitor cytokine profiles and C-peptides as markers of reduced inflammation and insulin synthesis recovery [101].

In summary, there is a critical need for future research to develop accessible and affordable diagnostic tests for autoimmune T1D, as early diagnosis significantly improves patient and family quality of life. Advances in genome, transcriptome, proteome, metabolome, and epigenome assessment have enabled identification of new T1D biomarkers, improved understanding of its pathogenesis, and heralded the era of precision medicine. Elevating the precision of detection of subjects with Stage 0 T1D will allow the implementation of more effective screening programs and mitigate such adverse events as the risk of overdiagnosis or the psychological impact of preclinical labeling. Integrative multiomic approaches for diabetes and its complications permit precise diagnostics of disease mechanisms and subtypes, and may provide new strategies for prediction, classification, and personalized treatment and lower economical burden of symptomatic therapy of T1D and its complications. The most promising of the prospective markers approach for the earliest T1D risk assessment is the use of polygenic risk scores, which are not yet widely used in clinical practice because of the lack of clear protocols and standards. It is important to note the difficulty in assessing PGS transferability across ethnicities, which researchers will need to address in the development of genetic risk panels. Improvements will require additional studies with larger T1D cohorts to expand current databases and deployment of machine learning and artificial intelligence for their integration. PGS will allow for the assessment of predisposition. At the stage of the beginning of beta-cell destruction, the assessment of cfDNA and microRNA levels may be useful, but there are no uniform protocols for the diagnosis of T1D for these biomarkers.

Preclinical T1D diagnostics was and still remains a complicated scientific and clinical task. Analysis of the existing T1D markers shows that solving the task of revealing Stage 1 of the disease is achievable for modern science under the condition that the studies will be continued and a complex panel of early autoimmune processes will be elaborated on the base of the data on cfDNA, miRNA, cytokines, and T1D-specific immune cells (including islet-specific T-cells). Revealing Stage 0 is a more complex task. Nevertheless, new discoveries in studies of immune and genetic heterogeneity of T1D and determination of the disease’s endotype will probably be finalized by the development of reasonably optimal screening programs with low overdiagnosis share. However, due to the importance of trigger factor action for T1D progression, 100% accuracy of revealing Stage 0 patients sounds more like a utopia. Implementation of novel diagnostic measures should be conducted with strict consideration of ethical and legal norms with voluntary consent of the patients.

Thus, preclinical T1D diagnostics, in our opinion, should aim to assign a risk group for each individual and to estimate the likely age of onset and T1D phenotype. Further work with the revealed risk groups requires developing an optimized surveillance scheme (based thereon) and, at each step, profile autoimmune process markers and targets for potential therapeutic options. The version of a personalized approach to diagnostics and therapy of this disease may be the elaboration of several early diagnostics panels or a universal panel with variable components depending on the immunotype of T1D patients. To realize it, panels with a wide range of T1D markers should be designed after retrospective and prospective studies of T1D markers in large cohorts taking into account biological differences and the possibility of immunotype assignment in both control groups and groups with high risk, prediabetes, and T1D. A coordinated effort on unification of standard operational procedures and analytical approaches should be conducted. Moreover, before implementation to the clinical practice, the proposed scales should be standardized considering the variability between analytical methods, preanalytical effects and risk of false-positive results [183]. It should also be taken into account that advanced technologies such as blood scRNA-seq could allow the definition of even finer and deeper T1D markers, but are not yet feasible in large-scale routine diagnosis due to cost and complexity of interpretation for treatment benefit. Also, the detection of IAR T lymphocytes currently requires optimization of the technologies of T1D-specific clones, expansion, and elaboration of the public T1D TCR database, and lowering of the costs of analyses [184]. Increased awareness and dissemination of new T1D staging criteria among primary care providers is also essential, since their vigilance determines the recognition of potential cases and referral for diagnosis. Finally, strengthening partnerships between laboratories and clinicians, as well as between primary and secondary care physicians, is critically important.

## Figures and Tables

**Table 1:** Primary HLA haplotypes and their effect on T1D.

Haplotype	Effect	Hetero/Homo	Source
HLA-DQA1*05:01/DQB1*02:01	Susceptibility	-	[20]
HLA-DRA1*01:01/DRB1*04:01	Susceptibility	-	[20]
HLA-DRA1*01:01/DRB1*04:05	Susceptibility	-	[20]
HLA-DQA1*03:01/DQB1*03:02	Susceptibility	both	[21]
HLA-DRB1*03:04	Susceptibility	both	[21]
HLA-DRB1*03:01	Susceptibility	both	[21]
HLA-DRB1*04:04	Susceptibility	both	[21]
HLA-DRB1*04:01	Susceptibility	both	[21]
HLA-DRB1*08:01	Susceptibility	-	[22]
HLA-DPA1*01:03/DPB1*03:01	Susceptibility	-	[23]
HLA-DRB1*08:01/DQA1*04:01/DQB1*04:02	Susceptibility	-	[24]
HLA-DRB1*04:01/DQA1*03/DQB1*03:02	Susceptibility	-	[25]
HLA-DRB1*03:01/DQA1*05:01/DQB1*02:01	Susceptibility	-	[13]
HLA-DRB1*04:05	Susceptibility	-	[13]
HLA-DRB1*09:01/DQB1*03:03	Susceptibility	homo	[26]
HLA-DRB1*04:05/DQB1*04:01	Susceptibility	hetero	[26]
HLA-DRB1*08:02/DQB1*03:02	Susceptibility	hetero	[26]
HLA-DRB1*04:04/DQA1*03:01/DQB1*03:02	Susceptibility	-	[25]
HLA-DRB1*04:05/DQA1*03:01/DQB1*03:02	Susceptibility	-	[13]
HLA-DRB1*04:01/DQA1*03:01/DQB1*03:02	Susceptibility	-	[13]
HLA-DRB1*04:02/DQA1*03:01/DQB1*03:02	Susceptibility	-	[13]
HLA-DRB1*03:01/DQA1*05:01/DQB1*02:01	Susceptibility	-	[13]
HLA-DRA1*01:01/DRB1*04:03	Protection	-	[20]
HLA-DQA1*01:02/DQB1*06:02	Protection	-	[20]
HLA-DPA1*01:03/DPB1*04:02	Protection	-	[23]
HLA-DPA1*01:03/DPB1*01:01	Protection	-	[23]
HLA-DRB1*15:01	Protection	-	[27]
HLA-DRB1*13:03/DQA1*05:01/DQB1*03:01	Protection	-	[13]
HLA-DRB1*11:04/DQA1*05:01/DQB1*03:01	Protection	-	[13]
HLA-DRB1*15:01/DQA1*01:02/DQB1*06:02	Protection	-	[13]
HLA-DRB1*07:01/DQA1*02:01/DQB1*03:03	Protection	-	[13]
HLA-DRB1*14:01/DQA1*01:01/DQB1*05:03	Protection	-	[13]
HLA-DQB1*06:02	Protection	-	[25]

**Table 2:** Non-HLA SNPs effect on the T1D disease process.

SNP	Gene	Gene Product Function	T1D Patients/ Control, N	Population According to Author Data	Sex, Age	Source
rs17885785	INS	Insulin is involved in carbohydrate metabolism regulation	1590/10,718	Canadian, Caucasians	Males and females, 3–17 years, with a mean age of onset of 7.9 years	[28]
rs7795896	CFTR	Cystic fibrosis transmembrane regulator involved in the transport of chloride ions across the cell membrane	18,942/501,638	European ancestry, Caucasians	Males and females, exact ages not specified	[29]
rs2269241	PGM1	Phosphoglucomutase 1 catalyzes the transfer of phosphate between positions 1 and 6 of glucose	25,193/35,476	European, African American, East Asian, Finnish, mixed ancestry	Males and females, exact ages not specified	[30]
rs34090353	RPAP2	RNA polymerase II-associated protein 2 is involved in dephosphorylation of the RNA polymerase II C-terminal domain and snRNA transcription	25,193/35,476	European, African American, East Asian, Finnish, mixed ancestry	Males and females, exact ages not specified	[30]
rs2229238	IL6R	Subunit of the IL6 receptor complex, regulator of IL6 signaling pathways	25,193/35,476	European, African American, East Asian, Finnish, mixed ancestry	Males and females, exact ages not specified	[30]
rs2816313	RGS1	Regulator of G protein signaling 1, a critical mediator of T-cell regulatory function	25,193/35,476	European, African American, East Asian, Finnish, mixed ancestry	Males and females, exact ages not specified	[30]
rs11120029	TATDN3	TATDN3 protein provides metal ion binding activity and nuclease activity	25,193/35,476	European, African American, East Asian, Finnish, mixed ancestry	Males and females, exact ages not specified	[30]
rs10169963	AC096559.1	Non-coding RNA	25,193/35,476	European, African American, East Asian, Finnish, mixed ancestry	Males and females, exact ages not specified	[30]
rs12712067	AFF3	Protein AFF3—nuclear transcriptional activator of lymphoid tissue	25,193/35,476	European, African American, East Asian, Finnish, mixed ancestry	Males and females, exact ages not specified	[30]
rs10933559	FARP2	Provides activity of guanine nucleotide exchange factor	25,193/35,476	European, African American, East Asian, Finnish, mixed ancestry	Males and females, exact ages not specified	[28]
rs1876142	PTGER4	Prostaglandin E2 (PGE2) receptor, participates in T-cell activation	25,193/35,476	European, African American, East Asian, Finnish, mixed ancestry	Males and females, exact ages not specified	[29]
rs9405661	IRF4	Interferon regulatory factor 4, plays an important role in antiviral responses and in the regulation of interferon-induced genes	25,193/35,476	European, African American, East Asian, Finnish, mixed ancestry	Males and females, exact ages not specified	[30]
rs12665429	TNFAIP3	Possesses both ubiquitin ligase and deubiquitinase activity, inhibits the activation of transcription factors NF-kB and AP-1 and cytokine-induced apoptosis	25,193/35,476	European, African American, East Asian, Finnish, mixed ancestry	Males and females, exact ages not specified	[30]
rs17143056	ABCB5	Involved in ATP-dependent transmembrane transport of structurally diverse molecules	25,193/35,476	European, African American, East Asian, Finnish, mixed ancestry	Males and females, exact ages not specified	[30]
rs2250903	CTSB	Cathepsin B, lysosomal cysteine protease with both endopeptidase and exopeptidase activity	25,193/35,476	European, African American, East Asian, Finnish, mixed ancestry	Males and females, exact ages not specified	[30]
rs1405209	NR4A3	Transcriptional activator	25,193/35,476	European, African American, East Asian, Finnish, mixed ancestry	Males and females, exact ages not specified	[30]
rs722988	NRP1	Neuropilin 1, mediates insulin signaling pathways, involved in signaling pathways controlling cell migration	25,193/35,476	European, African American, East Asian, Finnish, mixed ancestry	Males and females, exact ages not specified	[30]
rs79538630	CD5/CD6	CD5 and CD6 are receptors at the interface of the innate and adaptive immune responses of T-lymphocytes, involved in cell adhesion and important for the continued activation of T-cells	25,193/35,476	European, African American, East Asian, Finnish, mixed ancestry	Males and females, exact ages not specified	[30]
rs645078	CCDC88B	Protein CCDC88B, involved in the binding of organelles to microtubules	25,193/35,476	European, African American, East Asian, Finnish, mixed ancestry	Males and females, exact ages not specified	[30]
rs605093	FLI1	Transcription factor containing an ETS DNA-binding domain	25,193/35,476	European, African American, East Asian, Finnish, mixed ancestry	Males and females, exact ages not specified	[30]
rs7313065	ITGB7	Integrin beta-7, involved in signaling from the extracellular matrix to the cell, migration of lymphocytes to the intestine	25,193/35,476	European, African American, East Asian, Finnish, mixed ancestry	Males and females, exact ages not specified	[30]
rs74537115	AKAP11	Anchor proteins of A-kinase are involved in binding to the regulatory subunit of protein kinase A and localization of holoenzyme within the cell; participant in the cell cycle control system	25,193/35,476	European, African American, East Asian, Finnish, mixed ancestry	Males and females, exact ages not specified	[30]
rs4238595	UMOD	Uromodulin, inhibitor of calcium crystallization in renal fluids	25,193/35,476	European, African American, East Asian, Finnish, mixed ancestry	Males and females, exact ages not specified	[30]
rs2597169	PRR15L	Proline-rich 15-like protein: associated with pancreatic cancer subtypes	25,193/35,476	European, African American, East Asian, Finnish, mixed ancestry	Males and females, exact ages not specified	[30]
rs56178904	ICOSLG	Involved in the T-cell receptor signaling pathway and positive regulation of interleukin-4 production	25,193/35,476	European, African American, East Asian, Finnish, mixed ancestry	Males and females, exact ages not specified	[30]
rs689	INS	Insulin involved in carbohydrate metabolism regulation	3532/3607	African, European, mixed descent	Males and females, exact ages not specified	[24]
rs6679677	PTPN22	Non-receptor protein tyrosine phosphatase 22 involved in regulating CBL function in T-cell receptor signaling pathway, T-cell inhibitor	3532/3607	African, European, mixed descent	Males and females, exact ages not specified	[24]
rs61839660	IL2RA	IL2 receptor alpha subunit, regulator of immune functions of regulatory T-cells	3532/3607	African, European, mixed descent	Males and females, exact ages not specified	[24]
rs737391	RNLS	Renalase provides binding activities for NADH, adrenaline, and monoamine oxidase	3532/3607	African, European, mixed descent	Males and females, exact ages not specified	[24]
rs7302200	IKZF4- RPS26- ERBB3	Intergenic variant	3532/3607	African, European, mixed descent	Males and females, exact ages not specified	[24]
rs597808	SH2B3	Adapter protein encoded by the human gene SH2B3, a key negative regulator of cytokine signaling	3532/3607	African, European, mixed descent	Males and females, exact ages not specified	[24]

**Table 5:** Analysis of the T1D association with the HLA region in different populations.

Population	Allele		Haplotype		Reference
	Increased Risk	Decreased Risk	Increased Risk	Decreased Risk	
Arabs	DRB1*03:01, DRB1*04:02, DQB1*02:01, DQB1*03:02	DRB1*11:01, DRB1*16:02, DQB1*03:01, DQB1*06:01	DRB1*03:01DQB1*02:01, DRB1*04:02DQB1*03:02, DRB1*04:05- DQB1*03:02	DRB1*16:02DQB1*05:02	[55]
Africans	DQA1*03:01	—	HLA-DRB1*03:01- DQA1*05:01- DQB1*02:01	—	[24]
Bahrainis	DRB1*03:01:01, DRB1*04:01:01, DQB1*02:01, DQB1*03:02	DRB1*11:01:01, DQB1*03:01, DQB1*05:01:01	DRB1*03:01:01- DQB1*02:01, DRB1*04:01:01- DQB1*03:02	DRB1*10:01:01DQB1*05:01:01	[56]
Brazilians	DRB1*03, DRB1*04, DQA1*03:01, DQA1*03:02, DQA1*05:03, DQA1*05:05	DRB1*08, DRB1*11, DRB1*13, DRB1*14, DRB1*15, DQA1*01:01, DQA1*01:02, DQA1*01:03, DQA1*04:01	DRB1*03:01 DQA1*05:01- DQB1*02:01	—	[57]
Buryats	DRB1*04	DRB1*01, DRB1*11, DRB1*13, DRB1*15	—	—	[58]
Europeans	DQB1*03:02	—	HLA-DRB1*04:01- DQA1*03:01DQB1*03:02, HLA-DRB1*08:01- DQA1*04:01- DQB1*04:02	—	[24]
Jordanians	DRB1*04, DRB1*03:01, DQA1*03:01, DQA1*05:01, DQB1*02:01, DQB1*03:02	DRB1*07:01, DRB1*11:01, DQA1*05:05, DQA1*01:03, DQA1*02:01, DQB1*03:01, DQB1*05:01	DRB1*04DQA1*03:01- DQB1*03:02, DRB1*03:01DQA1*05:01- DQB1*02:01	DRB1*11:01DQA1*05:05- DQB1*03:01	[59]
Iranians	DRB1*04:01, DRB1*03:01, DQB1*03:02, DQB1*02:01	DRB1*15:01, DRB1*:01, DQB1*03:01, DQB1*06:01	DRB1*04:01DQB1*03:02, DRB1*03:01DQB1*02:01, DRB1*07:01- DQB1*03:03	DRB1*15:01DQB1*06:01, DRB1*11:01- DQB1*03:01	[60]
Kalmyks	DRB1*09	DRB1*07, DRB1*11, DRB1*15	—	—	[58]
Lebanese	DRB1*03:01:01, DRB1*13:07:01, DQB1*02:01	DRB1*11:01:01, DQB1*03:01, DQB1*05:01:01	DRB1*03:01:01 DQB1*02:01	DRB1*15:01:01 DQB1*06:01:01	[56]
Mari	DRB1*03, DRB1*04	DRB1*07, DRB1*11, DRB1*13, DRB1*15	—	—	[58]
Russians	DRB1*03, DRB1*04	DRB1*07, DRB1*11, DRB1*13, DRB1*15, DRB1*16	—	—	[58]
Tatars	DRB1*01, DRB1*03, DRB1*04	DRB1*07, DRB1*13, DRB1*15	—	—	[58]
Tunisians	DRB1*04:01:01	DRB1*11:01:01, DQB1*03:01:01, DQB1*06:01:01	DRB1*03:01:01- DQB1*02:01, DRB1*04:01:01 DQB1*03:02	—	[56]
Tuvans	DRB1*03	DRB1*13, DRB1*15	—	—	[58]
Udmurts	DRB1*01, DRB1*03, DRB1*04	DRB1*11, DRB1*13, DRB1*15	—	—	[58]
Uzbeks	DRB1*03, DRB1*04	DRB1*07, DRB1*13, DRB1*15	—	—	[58]
Swedes	DRB1*04:01, DRB1*04:02, DRB1*04:04, DRB1*04:05	DRB1*04:03, DRB1*04:07	—	—	[61]
Japanese	HLA-B*54:01, HLA-A amino acid position 62	—	—	—	[62]

**Table 6:** Functions of certain cytokines.

Cytokine	Function	Reference
IL-1	Causes dysfunction and death of *β*-cells.	[84]
IL-6	Serves as a key regulator of the migration and inflammatory responses of effector T and B cells.	[85]
TNF-*α*	Enhances the expression of MHC-I molecules, thereby accelerating antigen presentation and apoptosis of *β*-cells, exhibiting direct cytotoxic effects.	[80]
IFN-1	Induces increased presentation of autoantigens by islet cells, thereby enhancing the activation of effector T-cells.	[81]
IFN-*α*	Facilitates the presentation of self-antigens by islet cells, leading to recognition of these cells by cytotoxic T-lymphocytes. Induces the secretion of various chemokines involved in the recruitment of immune cells, such as T and NK lymphocytes and provokes oxidative stress.	[86,87]
IFN-*γ*	Mediates the destruction of *β*-cells in local islets and induces aberrant expression of MHC-I and MHC-II in local pancreatic cells, resulting in autoimmune *β*-cell death.	[88]
IL-17	Through the IL-17RA and RC receptor complex, which is widely present on the surface of islet cells, IL-17A exacerbates islet inflammation by directly inducing apoptosis of *β*-cells and locally increasing levels of pro-inflammatory cytokines and chemokines. In interaction with IFN-*γ* and IL-1*β*, it synergistically induces inflammation and apoptosis in human pancreatic islet cells.	[89]
IL-2	Exerts pleiotropic effects on various immune cell populations, including NK cells, effector T-cells, and Tregs.	[90]
IL-4	Participates in the activation of the PI3K and JAK/STAT pathways, contributing to the viability of insulin-producing cells, as well as stimulating IL-2 synthesis and the activation and expansion of iNKT and Treg cells.	[91]
IL-13	Activates the STAT signaling pathway, suppressing the ongoing destruction of *β*-cells and preventing the development of T1D.	[92]
IL-10	Induces an increase in the number of Tregs, elevates levels of Th2-type cytokines (IL-4 and IL-10), and reduces the Th1 response (IL-2 and IFN-*γ*). Moreover, IL-10 is associated with a tolerant state of immature dendritic cells (DCs) and Bregs in humans and mice with T1D, promoting insulin-specific tolerance in effector and memory T-cells generated in T1D patients.	[93,94]
TGF-*β*	Induces the expression of Foxp3 and the differentiation of peripheral Tregs.	[95,96]
HGF	The signaling of HGF/c-Met in *β*-cells is essential for normal growth and function of *β*-cells under basal conditions and is critically important for the survival of *β*-cells in diabetes.	[97]

**Table 8:** The application, prospects, and readiness level of the biomarker for implementation in clinical practice and preclinical diagnosis of T1D.

Biomarker	Research Only	Available Screening Assay	Guideline- Premorbid Supported Diagnostics		Early *β*-Cell Destruction Diagnostics	Preventive Therapy Choice	Cost- Effectiveness of Implementing Biomarker Panels *
			**Clinical biomarkers**				
Genetic (HLA-haplotype, Non-HLA SNPs, PGS)	-	+ [5,31,38]	+ [38,178]	Yes, overdiagnosis [38,178]	No	Potential [38,178]	+
Islet Antigen Autoantibodies	-	+ [7,178,179,180]	+ [7,178,179,180]	No	Yes, overdiagnosis [7]	Potential [7]	+++
C-Peptide	-	+ [7,178]	+ [7,178]	No	No	No	+++
			**Exploratory biomarkers**				
Cytokines	+ [101]	+ [178]	-	No	Potential [81]	Potential [114,115]	+++
cfDNA	+ [130,135,136,137,138]	-	-	No	Potential [129,130]	Potential [129,130]	++ [133,134,137]
MicroRNA	+ [156]	-	-	No	Potential [149]	Potential [148]	+ [147,157,158]
T1D specific immune cells	+ [166,167]	-	-	Potential [163]	Potential [164,165]	Potential [181,182]	+ [29]
Islet-TCR	+ [167]	-	-	Potential [166]	No	Potential [168,169]	+
T1D specific vibrational bands	+ [174,175,176]	-	-	Potential [175,176]	Potential [175,176]	Potential [177]	+++

* + is low cost, ++ is medium cost and +++ is high cost.

## Data Availability

Not applicable.
